# Difference in left renal vein pressure: an indicator for free of reconstruction after ligation in retroperitoneal tumor patients

**DOI:** 10.1038/srep18126

**Published:** 2015-12-11

**Authors:** Chengli Miao, Mengmeng Xiao, Tengyan Li, Gang Liu, Xing Liu, Yue Kong, Chenghua Luo

**Affiliations:** 1 Department of Retroperitoneal Tumors Surgery Peking University International Hospital, Beijing, P.R. China; 2 Department of Surgical Oncology, Beijing Shijitan Hospital, Capital Medical University (The 9th affiliated hospital of Peking University), Beijing, P.R. China; 3 Ultrasound Department, Beijing Shijitan Hospital, Capital Medical University (The 9th affiliated hospital of Peking University), Beijing, P.R. China

## Abstract

We hypothesized that the left renal vein pressure difference (ΔP) before and after the ligation can serve as an objective indicator for free of reconstruction after resection of a retroperitoneal tumor with renal segment of inferior vena cava and right kidney. After established a model of left renal vein compression, 45 miniature pigs were operated on experimental procedures including renal segment of inferior vena cava resection, right nephrectomy, and left renal vein ligation. The ΔPs of left renal vein before and after the ligation were measured. Safe ΔP variation without causing acute kidney injury was calculated using regression analysis. In human the safety range of ΔP before and after ligation of the left renal vein was calculated by diuretic response test. The safety range of ΔP in animals or human was 0–11.9 or 0–17.5 cm H_2_O, respectively. The renal function changed dramatically (*p* < 0.01), characterized by a significant increase in the rate of acute kidney injury when the ΔP was beyond the upper limit of the safety range. In conclusion, ΔP can predict free of reconstruction after resection of a retroperitoneal tumor with the renal segment of the inferior vena cava and the right kidney.

The retroperitoneum has been observed to host a wide spectrum of diseases, including a variety of rare benign tumors and malignant neoplasms that can be either primary or metastatic lesions. Are troperitoneal tumor (RPT)develops inside part of the abdominal cavity known as the retroperitoneal space. It is relatively uncommon in clinics, and about 60–85% of the RPT is malignant^1^,^2^,^3^. Importantly, RPTs can cause diagnostic dilemmas and therapeutic challenges due to their rarity, relative late presentation of symptoms, and anatomical location, often in close relationship with several vital structures in the retroperitoneal space. The vast majority of RPTs is refractory to chemotherapy and radiotherapy. Surgery is the only effective method of radical therapy^4^, therefore, a radical operation determines the prognosis of RPTs^5^.

At diagnosis, there always exists close relationships between the tumor body and important abdominal vasculatures, especially its invasion into the renal level and above the inferior vena cava. This situation very difficult to handle, which requires to rebuild the renal venous return, leading to an increase in the complexity of the operation as well as the incidence of operation-related complications. During the complicated RPT operation, artificial vascular or autogenous vascular reconstruction of inferior vena cava is extremely time-consuming and technically difficult. In addition, complications such as intraoperative hypothermia, acidosis and coagulation disorders would develop eventually. Furthermore, potential postoperative fatal pulmonary embolism also increases the patient’s mortality risk. Thus, we aimed to establish an animal model to explore the feasibility of conducting optimized operations on complicated RPT.

Based on the above challenge and difficulty in clinics, researchers divided the inferior vena cava below the hepatic venous level into three anatomic segments: Level I: from the iliac vessels to 2 cm below the renal vein level; Level II: from 2 cm below the renal vein level to 2 cm above renal vein level; and Level III: from 2 cm above the renal vein level to the level of hepatic vein. Level I and III, when invaded by tumors, can be simply resected in combination with vasculatures. Under this circumstance, there is no need to rebuild vasculatures because double renal veins can flow back through the internal iliac vein collateral circulation, without causing renal failure. When Level II has been invaded, if a complete occlusion and collateral circulation has been fully established, combined vasculature resection can be applied. There is no need to reconstruct vessels for it won’t cause renal failure^6^. Since the right renal vein collateral circulation has less blood volume, whereas the left kidney collateral circulation is rich in blood, collecting 3branches,include the adrenal vein, genital gland vein and lumbar veins^7^.

We believe that tumors invaded to Level II can be resected in combination with the right kidney, to prevent the necrosis of the right kidney and to reduce toxins produced by it. When the tumor has invaded the Level II inferior vena cava and made partial blockage, renal venous collateral circulation has not been fully established. Therefore, how to determine whether it is necessary to rebuild vasculature remains to be elucidated. To identify an objective index for free of reconstruction, we hypothesize that venous pressure difference (ΔP) before and after renal vein ligation can indirectly reflect the degree of collateral circulation, and thus serve as an objective indicator for free of reconstruction.

Tumor-induced compression of the inferior vena cava can result in blockage of renal veins, where the blood reflows through the inferior vena cava as a portion of the collateral circulation^8^. But after the ligation of renal vein, obstruction of blood flow through inferior vena cava will raise the renal vein pressure, and the change in the pressure (ΔP) of renal vein can reflect the presence of collateral circulation. We propose that there is a positively proportional relationship between ΔP value and the degree of renal injury. The most common operation mode is the resection of level II inferior vena cava and the right kidney, plus ligation of the left renal vein. Here we would emphasize a safe ΔP range during the operation.

## Materials and Methods

Our work flow was to 1) establish an animal model; 2) simulate an RPT; 3) mimic the blockage of the inferior vena cava at and above the kidney level; 4) establish the left renal vein blood reflux of collateral circulation; 5) implement resection of inferior vena level at and above renal vein level and the right kidney; and 6) operate on the left renal vein ligation. We detected the change of pressure in the left renal vein during the operation, investigated whether the pressure difference (ΔP) could be used as an objective indicator to determine free of reconstruction of left renal vein and inferior vena venous reflux pathway, and finally analyzed its applicability in clinics.

### Animals

45 Gui zhou miniature pigs (22 male and 23 female) aged of 5 months-old and weighed at 10–15 kg were purchased from Beijing Liuli Sinica experimental animal breeding center, Beijing Academy of Science and Technology. This animal study was approved by the Institutional Animal Care and Use Committee of Beijing Shi ji tan Hospital, and the experimental methods were carried out in accordance with the approved guidelines.

### Patients

Totally 25 patients with RPTs were recruited at Beijing Shijitan Hospital from January 2012 to August 2013 and follow up for at least 2 years. Written informed consent for an interview and a sample donation was obtained from each study participant. The study was approved by the Institutional Review Board of Beijing Shijitan Hospital and was conducted in accordance with all current ethical guidelines.

### Measurement of left renal vein pressure

**Figure 1:**
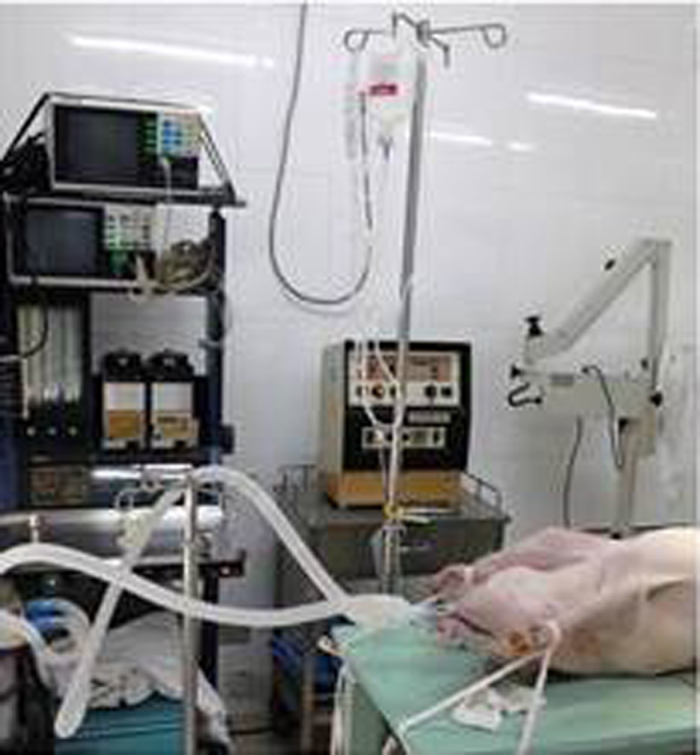
General Anesthesia for the miniature pig.

**Figure 2:**
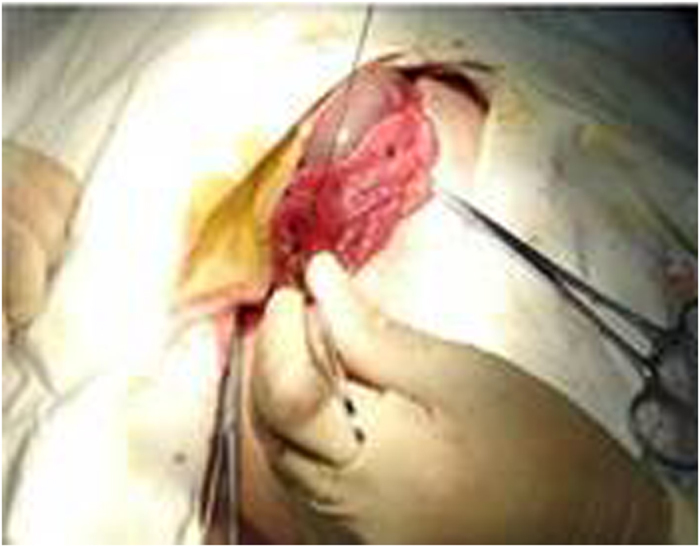
Anatomy of the pig’s left renal vein. A circled silicone sling was placed around the vein.

**Figure 3:**
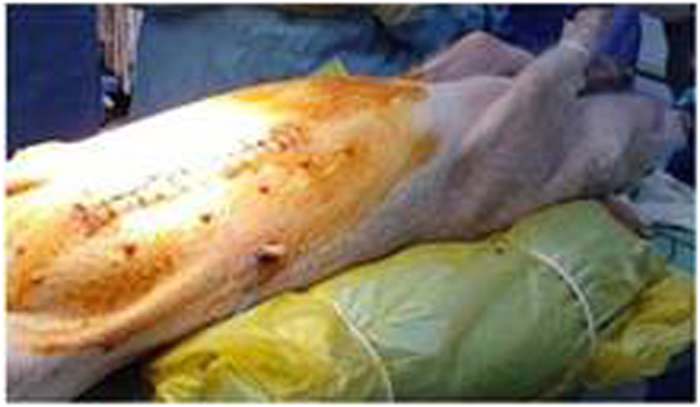
Established animal model of the left renal vein blood reflux of collateral circulation.

**Figure 4:**
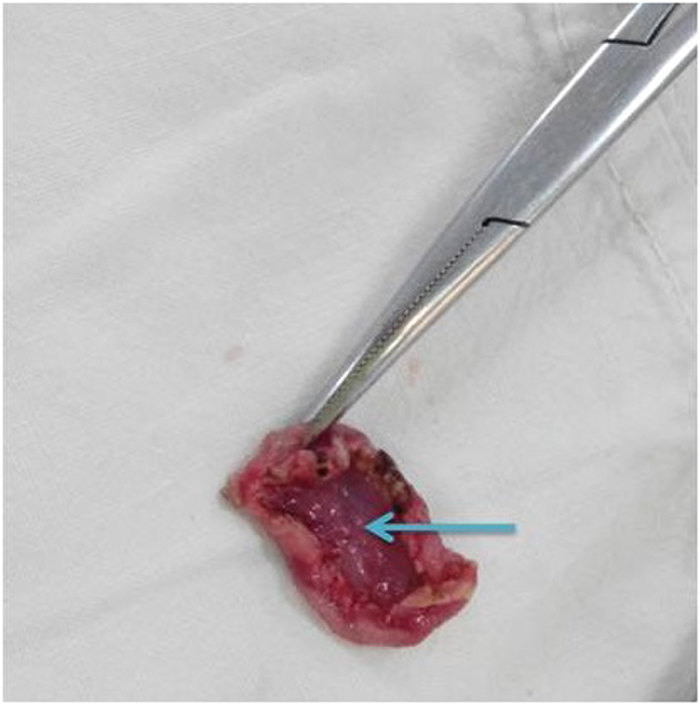
Resected pig’s inferior vena cava. The vasculature had been cut,with the arrow pointing to vessel lumen.

**Figure 5:**
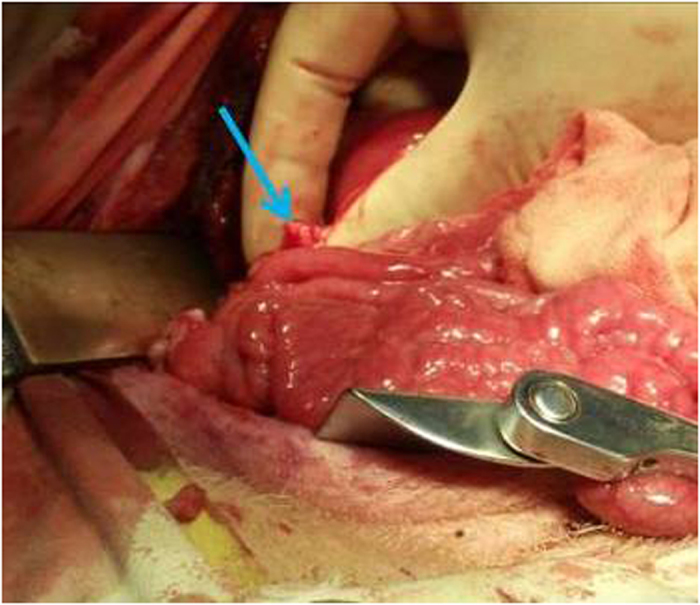
Ligature of the pig’s left renal vein. Blue arrow pointed to the renal artery, which had been ligated during the surgery.

**Figure 6:**
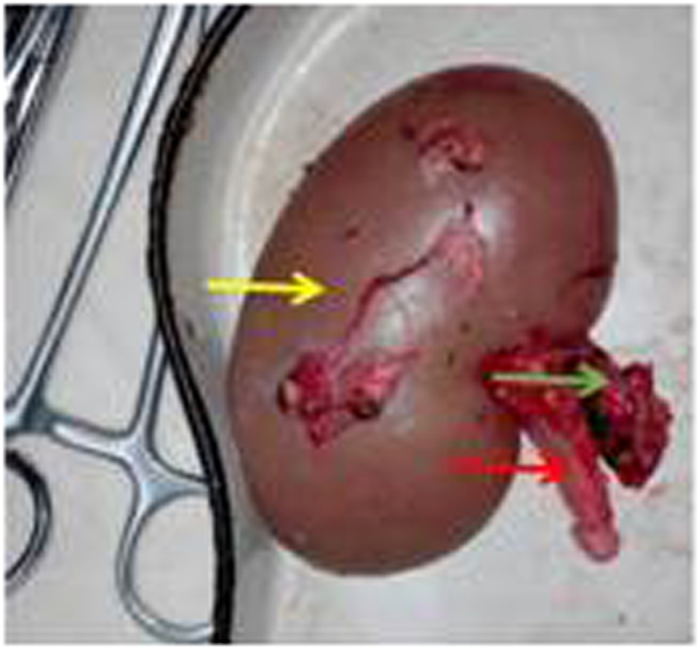
Resected pig’s right kidney(yellow). Red arrowpointed to the renal artery, and the green arrow pointed to the ureter.

We simulated different degrees of tumor compression induced left renal vein reflux obstacle in the animal model. In brief, as shown in Fig. 1, under general anesthesia, the animals were in right lateral position. As shown in Figs 2, 3, 4, 5, 6, we took a left subcostal arc incision, cut subcutaneous tissue of the skin and muscle layer by layer, into the extraperitoneal space. We dissected bluntly to the left kidney, and made the entrance part of left renal vein free from the inferior vena cava. We circled silicone sling around vein, pulled two free ends of the sling from the hard tube drainage opening, guided the drainage pipe as well as the sling out of abdominal wall port, sutured pipe to the skin, and then bond the sling to the pipe. The wound was closed in layers at the end of operation. The silicone sling would be gradually tightened postoperatively, and then the left renal vein blood flow would decrease to different extent, which could be measured by vascular ultrasonography. Totally 25 miniature pigs were divided into 5 groups (A: blood flow decreased by 0%, serving as the control; B: blood flow decreased by 25%; C: blood flow decreased by 50%; D: blood flow decreased by 75%; and E: blood flow decreased by 100%). After the model was established, we implemented resection of inferior vena cava at and above renal vein level, the right nephrectomy, and ligation operation of left renal vein. The pressure changes (ΔP) of left renal vein before and after ligation was measured. Glomerular filtration rate (GFR) describes the flow rate of filtered fluid through the kidney. Creatinine clearance rate (CCr) is the volume of blood plasma that is cleared of creatinine per unit time and is a useful measure for approximating the GFR. Normal renal function was defined as free of acute kidney injury^9^. Acute kidney injury was defined when the blood creatinine increased by more than 50% from the standard basis within 48 hours after operation.

### Statistical analysis

We used SPSS17.0 software to carry out regression analysis. All results were expressed as mean ± SD. Statistical analyses were performed with the two-tailed Student’s t-test or Wilcoxon rank sum test between control and treatment groups. Due to the normal distribution of the results, comparison of pressure change before and after treatment was evaluated by paired t-test. A *p* value of < 0.05 was considered statistically significant.

## Results

### Renal function evaluation

The renal function represented by CCr and ΔP before and after operation was evaluated as shown in Table 1.The increased percentage of creatinine was considered as an independent variable, whereas ΔP as the dependent variable. The result for line regression analysis with one unknown quantity was: the regression coefficient = 0.027, constant = 10.517, and the regression equation: Y = 10.517 + 0.027X. Here, X = 50 was the critical indicator of acute kidney injury. Then, P = 10.517 + 0.027 × 50 = 11.867 (H_2_O cm). Thus, the safe range of ΔP value was from 0 to 11.9 cm H_2_O. Then, a random control study was designed as the following: The animals were randomly divided into two groups based on the ΔP values either within (≤11.9) or greater (>11.9) than the “safe range”. The relationship between ΔP value and change of renal function was evaluated. Totally 20 miniature pigs were made into models with reference to the first experimental design. As shown in Figs 2, the following operation was implemented: resection of inferior vena cava at and above renal vein level, the right nephrectomy, and ligation operation of left renal vein. The pressure change (ΔP) of left renal vein before and after ligation was measured. The renal function (CCr) and the ΔP values before and after operation were shown in Table 2. Notably, the renal function of ΔP greater > 11.9 cm H_2_O group was significantly decreased (P < 0.01, 2-sided Student’s t-test). Based on the independent samples t-test, ΔP ≤ 11.9 cmH_2_O group (n = 11) had an average increase rate increatinine of 17.6 ± 15.5%; in contrast, ΔP > 11.9 cm H_2_O group (n = 9) had an average increase rate increatinine of 70.3 ± 23.9% (*P* < 0.01).

### Human study

#### Analysis of ΔP value range for non-reconstruction

Inclusion criteria were as the following: (1) RPTs invaded to inferior vena cava at and above the renal vein level; and patients had indications of an operation; (2) Angiography indicated backflow obstacles of inferior vena cava at and above the renal vein level; and (3) Patients didn’t suffer from preoperative chronic kidney disease. Exclusion criteria were as the following: (1) Patients received any operation unrelated to the inferior vena cava; (2) The inferior vena cava side wall was resected partly and sutured immediately; and (3) The inferior vena cava below renal vein level was resected, and inferior vena cava at and above renal vein level was complete. Demographic and clinical characteristics of the study population was presented in Table 3.

**Figure 7:**
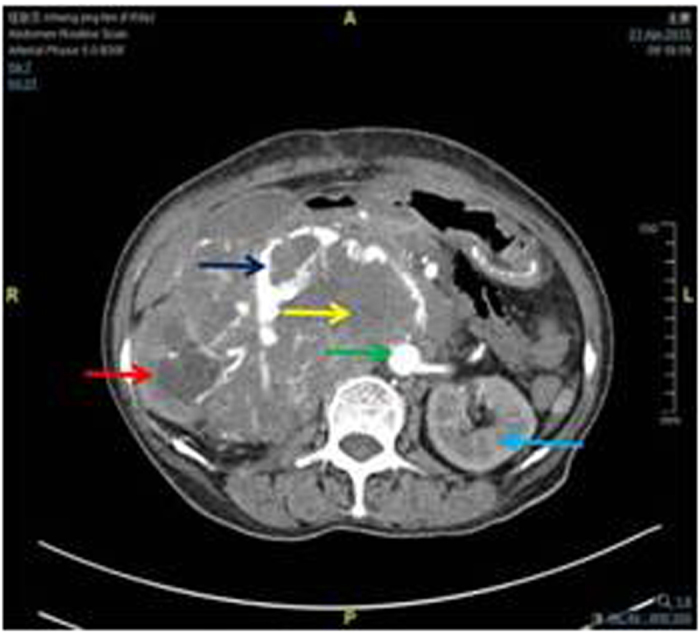
CT imaging of a patient with PRT. In this case, the tumor (yellow arrow) had invaded the inferior vena cava (level II, dark blue arrow), right kidney(red arrow) and left renal vein (blue arrow). The aorta abdominalis was highlighted by a green arrow.

**Figure 8:**
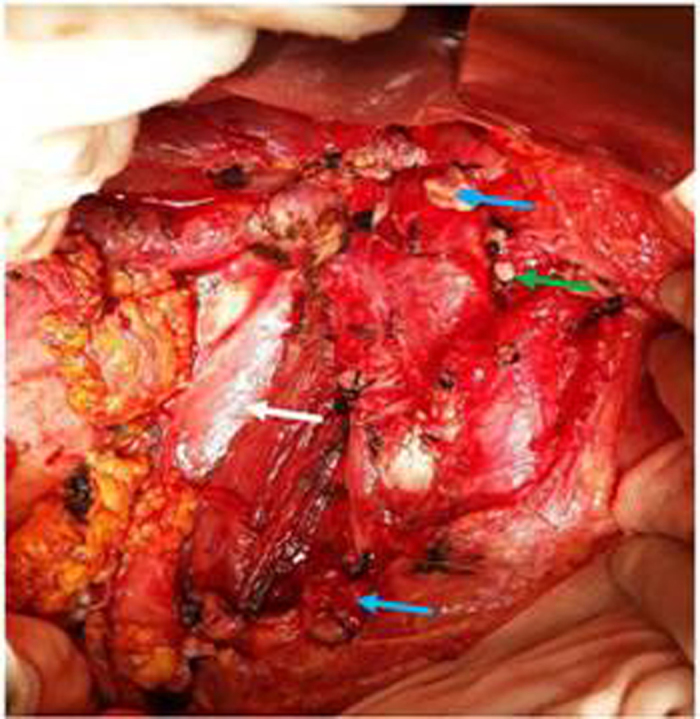
Resected patient’s inferior vena cava (blue arrow level II) was ligated at both ends. The right kidney(white arrow) and ligated left renal vein (green arrow) were shown.

**Figure 9:**
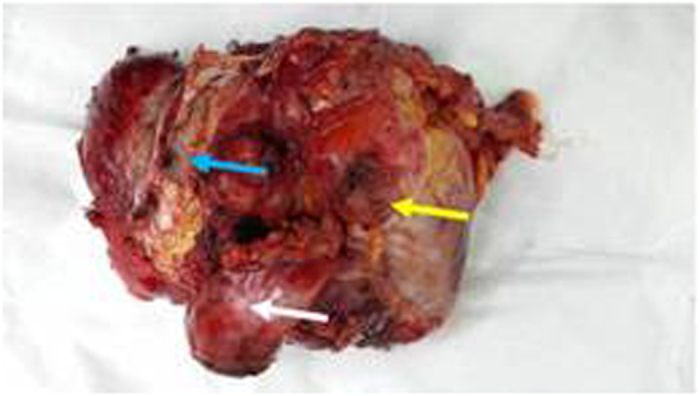
A representative resected patient specimen including the inferior vena cava(level II, blue arrow), the right kidney (white arrow)and the RPT (yellow arrow). The tumor had infiltrated the right kidney and the inferior vena cava.

A representative CT image of a patient with RPT was presented in Fig.7. As shown in Figs 8 and 9, the operation process included: the resection of the tumors, the right kidney, and the inferior vena cava at and above renal vein level, as well as the ligation of the left renal vein. Because the tumor had infiltrated to the right kidney and the inferior vena cava, we resected both of the organs and ligated the inferior vena cava at both ends. We measured ΔP values of the left renal vein before and after the ligation.The decision of reconstruction vs. non-reconstruction was made according to the diuretic response test. We blocked the right renal artery or ureter and ligated the left renal vein, intravenously injected furosemide 20–40 mg, and then observed the urine volume for 30 minutes. If the urine amount was greater than 50 ml, there was no need to reconstruct the left renal vein and inferior vena cava; if the urine volume is less than 50 ml, reconstruction was performed. Development of clinical operation in 25 cases was presented in Table 4.The urine volume in 30 minutes was considered as the independent variable, ΔP as the dependent variable, and the results for line regression analysis with one unknown quantity were as the following: the regression coefficient = −0.212, constant = 28.106. The regression equation was Y = 28.106 − 0.212X. Here, X = 50 was the critical value of the reconstruction of vessels; and then, ΔP = 28.106 − 0.212 × 50 = 17.506 cm H_2_O; therefore, the safe range of ΔP value was from 0 to 17.5 cm H_2_O.

## Discussion

In patients with complex RPTs where the inferior vena cava was invaded but not fully occluded at and above the renal level, we detected the changes in venous pressure before and after the ligation of the left renal vein. In accordance with the standard of the diuretic response test, we evaluated the necessity to reconstruct the left renal vein and inferior vena cava, and then analyzed the difference in ΔP value between the reconstruction vs. free of reconstruction group. For the patients free of reconstruction, no impairment of renal function was observed after operation, compared to the group with ΔP > 17.5 cm H_2_O who suffered from renal dysfunction. Therefore, we clarified the safe and feasible range of ΔP, which could serve as an objective and quantitative index for free of reconstruction.

Based on our findings, the pressure difference (ΔP) before and after the ligation of the left renal vein in patients with RPTs undergoing the resection of renal segment of inferior vena cava and the right nephrectomy, as well as diuretic response test, can be used to evaluate the degree of openness of lateral branches, so as to ensure the normal renal function after combine dresection. We have established an animal model using miniature pigs, and verified a positive correlation of ΔP with acute kidney injury. Using the linear regression analysis, we obtained the safe range of ΔP (0–11.9 cm H_2_O). Renal function from the group with ΔP > 11.9 was significantly reduced, and ΔP was an objective index for free of reconstruction. In human diuretics reaction test, after injection of furosemide, we obtained the safe range of ΔP (0–17.5 cm H_2_O). Thus, the animal model provided a reliable reference for human study, which may guide the treatment for those patients with RPTs.

It has been a clinical challenge to decide whether it is reasonable to resect the right kidney to avoid reconstruction of renal vasculature using the inferior vena cava. Such as retroperitoneal liposarcoma mainly originate from the perirenal fat tissue. To ensure a complete resection, we usually remove the tumors, perirenal fat tissue and kidney. Indeed, united resection can reduce the rate of local recurrence^10^. The majority of the kidney is infringed, in the case of RPTs invading into the renal vessels and the inferior vena cava. So combined resection is the best choice in most cases; while the prerequisite is the normal preoperative and postoperative contralateral renal function. Whether or not an orthotopic/heterotopic kidney transplantation should be performed, when the tumors simply invade into renal vascular and inferior vena cava. The operation duration for RPTs is usually long, and renal transplantation requires anatomy of vessels and ureter. The procedure will correspondingly increase the risk of thrombosis, poisoning, and postoperative bleeding. Thus, kidney transplant and artificial vascular operation will do more harm than benefit if the contralateral renal function is normal. The left renal vein is longer in size than the right one, containing the left gonadal vein, left lumbar vein, left adrenal vein and the left inferior phrenic vein as collateral circulation. The reflux of the left renal vein won’t be affected if it has been removed in most cases^2^. So the combined resection with the left renal vein is safe and feasible when the collateral circulation is fully open.

The angiography of the inferior vena cava also can be an indication of free for vascular reconstruction after combined resection of tumors, right kidney and inferior vena cava, besides the left renal vein pressure difference (ΔP) before and after the ligation, as well as the diuretic response test. Angiography or magnetic resonance angiography^11^ of inferior vena cava can obtain imaging evidence of the opening in the collateral circulation, however, quantification is impossible, so they can only estimate the degree of openness. Based on our animal and human studies, the left renal vein pressure difference (ΔP) is feasibly considered as an objective indicator for vasculature reconstruction. Considering a relatively small sample size may restrict universal applicability of this finding, we need to expand the data in a large patient population to validate the current conclusion. The measurement of pressure may also have a bias; For example, congestion and attachment to the vascular wall of the puncture needle may affect the result. We will improve the techniques in future experiments to obtain more accurate data.

## Additional Information

**How to cite this article**: Miao, C. *et al.* Difference in left renal vein pressure: an indicator for free of reconstruction after ligation in retroperitoneal tumor patients. *Sci. Rep.* **5**, 18126; doi: 10.1038/srep18126 (2015).

## Figures and Tables

**Table 1:** The relationship between pressure change of the left renal vein and acute kidney injury.

**ID**	**Basic creatinine (μmol/L)**	**Creatinine after modeling (μmol/L)**	**Creatinine at 24 hs Post-operative (μmol/L)**	**Proportion of increase in creatinine (%)**	**ΔP (H**_**2**_**O cm)**
pig1	63.0	69.0	74.0	7.2	2.0
pig2	50.0	51.0	138.0	170.6	37.5
pig3	50.0	49.0	84.0	71.4	15.0
pig4	53.0	66.0	788.0	1093.9	35.5
pig5	59.0	62.0	823.0	1227.4	40.4
pig6	36.0	50.0	62.0	24.0	7.1
pig7	47.0	66.0	109.0	65.2	26.3
pig8	66.0	85.0	125.0	47.1	15.4
pig9	55.0	59.0	82.0	38.9	10.4
pig10	49.0	83.0	136.0	63.9	32.1
pig11	51.0	62.0	94.0	51.7	13.5
pig12	50.0	65.0	70.0	7.7	2.6
pig13	49.0	34.0	41.0	20.6	5.1
pig14	59.0	45.0	51.0	13.3	5.0
pig15	65.0	59.0	62.0	5.1	0.0
pig16	51.0	66.0	66.0	0.0	0.0
pig17	50.0	63.0	67.0	6.3	0.0
pig18	47.0	58.0	66.0	13.8	7.2
pig19	47.0	57.0	66.0	15.8	7.3
pig20	52.0	50.0	48.0	−4	1.0
pig21	53.0	57.0	99.0	73.7	28.5
pig22	47.0	50.0	75.0	50.0	12.4
pig23	51.0	51.0	63.0	23.5	10.2
pig24	53.0	49.0	108.0	120.4	32.6
pig25	44.0	42.0	56.0	33.3	8.5

**Table 2:** Relationship between acute kidney injury and the change of P value of the left renal vein.

**ID**	**Basic creatinine (μmol/L)**	**Creatinine after modeling(μmol/L)**	**Creatinine at 24 hPost-operation (μmol/L)**	**Proportion of increase in creatinine (%) %**	**ΔP (cm H**_**2**_**O)**
pig26	41.0	38.0	59.0	55.3	12.3.
pig27	47.0	43.0	50.0	16.3	7.5
pig28	52.0	53.0	53.0	0.0	5.1
pig29	37.0	40.0	60.0	50.0	13.0
pig30	39.0	43.0	53.0	23.3	9.3
pig31	40.0	42.0	62.0	47.6	11.4
pig32	54.0	56.0	60.0	7.1	3.0
pig33	39.0	36.0	49.0	36.1	15.4
pig34	56.0	59.0	61.0	3.4	8.0
pig35	34.0	63.0	61.0	−3.2	0.0
pig36	58.0	51.0	83.0	62.7	13.4
pig37	60.0	65.0	89.0	36.9	4.3
pig38	55.0	44.0	83.0	88.6	25.6
pig39	32.0	43.0	69.0	60.5	5.3
pig40	41.0	44.0	92.0	109.1	32.5
pig41	34.0	43.0	84.0	95.3	35.1
pig42	44.0	43.0	69.0	60.5	19.0
pig43	56.0	58.0	69.0	19.0	7.0
pig44	61.0	54.0	64.0	18.5	7.0
pig45	42.0	39.0	70.0	79.5	17.6

**Table 3:** Demographic and Clinical characteristics of the study population.

**Index**	**Case (n = 25),%**
Age (years)	
≤60	13 (52.0)
>60	12 (48.0)
Gender	
Male	9 (36.0)
Female	16 (64.0)
Smoking	
Yes	10 (40.0)
No	15 (60.0)
Drinking	
Yes	11 (44.0)
No	14 (56.0)
Tumor size (cm)	
≤10	8 (32.0)
>10	17 (68.0)
Pathological pattern	
Leiomyosarcoma	18 (72.0)
Malignant fibrous histiocytoma	4 (16.0)
Liposarcoma	2 (8.0)
Malignant hemangiopericytoma	1(4.0)

**Table 4:** Urine volume in the diuretic response test and pressure changes.

**Patient ID**	**Diuretic response test (ml)**	**Reconstruction (Y/N)**	**ΔP(cm H**_**2**_**O)**
Patient1	44.5	Y	17.0
Patient2	25.0	Y	16.5
Patient3	84.5	N	9.2
Patient4	78.6	N	7.4
Patient5	9.5	N	7.3
Patient6	150.0	N	2.2
Patient7	109.0	N	4.0
Patient8	125.5	N	3.3
Patient9	32.5	Y	21.0
Patient10	13.5	Y	32.3
Patient11	45.3	Y	16.6
Patient12	10.0	Y	36.5
Patient13	41.0	Y	20.2
Patient14	51.5	N	16.4
Patient15	62.0	N	15.6
Patient16	66.4	N	18.2
Patient17	78.5	N	10.2
patient18	166.5	N	0.0
Patient19	106.3	N	1.0
Patient20	48.6	Y	19.4
Patient21	99.0	N	4.4
patient22	75.0	N	8.0
Patient23	63.5	N	14.2
Patient24	108.0	N	2.3
Patient25	36.3	Y	23.1

## References

[b1] Walgenbach S., Junginger T., Heuser L. & Pichlmaier H. [Primary retroperitoneal tumors. Symptoms, diagnosis and therapy]. Fortschr. Med. 101, 1897–1902 (1983). 6654289

[b2] Strauss D. C., Hayes A. J. & Thomas J. M. Retroperitoneal tumours: review of management. Ann. R. Coll. Surg. Engl. 93, 275–280 (2011). 21944791 10.1308/003588411X571944 PMC3363075

[b3] Osman S. *et al.* A comprehensive review of the retroperitoneal anatomy, neoplasms, and pattern of disease spread. Curr. Probl. Diagn. Radiol. 42, 191–208 (2013). 24070713 10.1067/j.cpradiol.2013.02.001

[b4] Mullinax J. E., Zager J. S. & Gonzalez R. J. Current diagnosis and management of retroperitoneal sarcoma. Cancer Control. 18, 177–187 (2011). 21666580 10.1177/107327481101800305

[b5] Ito F. *et al.* Combined resection of abdominal aorta and inferior vena cava for retroperitoneal rhabdomyosarcoma invading the aortoiliac bifurcation. J. Pediatr. Surg. 33, 1566–1568 (1998). 9802817 10.1016/s0022-3468(98)90501-2

[b6] Daylami R. *et al.* Inferior vena cava leiomyosarcoma: is reconstruction necessary after resection? J. Am. Coll. Surg. 210, 185–190 (2010). 20113938 10.1016/j.jamcollsurg.2009.10.010

[b7] Ye C. *et al.* Multislice computed tomographic angiography in evaluating dysfunction of the vascular access in hemodialysis patients. Nephron. Clin. Pract. 104, 94–100 (2006). 10.1159/000093996 16785741

[b8] Arkadopoulos N. *et al.* Inferior vena cava obstruction and collateral circulation as unusual manifestations of hepatobiliary cystadenocarcinoma. Hepatobiliary & pancreatic diseases international: Hepatobiliary. Pancreat. Dis. Int. 12, 329–331 (2013). 23742780 10.1016/s1499-3872(13)60052-1

[b9] Patschan D. & Muller G. A. Acute kidney injury. J. Inj. Violence Res. 7, 19–26 (2015). 25618438 10.5249/jivr.v7i1.604 PMC4288292

[b10] Gronchi A. *et al.* Frontline extended surgery is associated with improved survival in retroperitoneal low- to intermediate-grade soft tissue sarcomas. Ann. Oncol. 23, 1067–1073 (2012). 21765179 10.1093/annonc/mdr323

[b11] Corral D. A. *et al.* Magnetic resonance imaging and magnetic resonance angiography before postchemotherapy retroperitoneal lymph node dissection. Urology. 55, 262–266 (2000). 10688091 10.1016/s0090-4295(99)00428-8

